# Long-term exposure to air pollution and the incidence of Parkinson’s disease: A nested case-control study

**DOI:** 10.1371/journal.pone.0182834

**Published:** 2017-08-15

**Authors:** Chiu-Ying Chen, Hui-Jung Hung, Kuang-Hsi Chang, Chung Y. Hsu, Chih-Hsin Muo, Chon-Haw Tsai, Trong-Neng Wu

**Affiliations:** 1 Department of Public Health, China Medical University, Taichung, Taiwan; 2 Graduate Institute of Clinical Medical Science, College of Medicine, China Medical University, Taichung, Taiwan; 3 Management Office for Health Data, China Medical University Hospital, Taichung, Taiwan; 4 School of Medicine, College of Medicine, China Medical University, Taichung, Taiwan; 5 Department of Neurology, China Medical University Hospital, Taichung, Taiwan; 6 Department of Healthcare Administration, Asia University, Taichung, Taiwan; Liverpool School of Tropical Medicine, UNITED KINGDOM

## Abstract

**Background:**

Previous studies revealed that chronic exposure to air pollution can significantly increase the risk of the development of Parkinson’s disease (PD), but this relationship is inconclusive as large-scale prospective studies are limited and the results are inconsistent. Therefore, the purpose of this study was to ascertain the adverse health effects of air pollution exposure in a nationwide population using a longitudinal approach.

**Materials and methods:**

We conducted a nested case-control study using the National Health Insurance Research Dataset (NHIRD), which consisted of 1,000,000 beneficiaries in the National Health Insurance Program (NHI) in the year 2000 and their medical records from 1995 to 2013 and using public data on air pollution concentrations from monitoring stations across Taiwan released from the Environmental Protection Administration to identify people with ages ≥ 40 years living in areas with monitoring stations during 1995–1999 as study subjects. Then, we excluded subjects with PD, dementia, stroke and diabetes diagnosed before Jan. 1, 2000 and obtained 54,524 subjects to follow until Dec. 31, 2013. In this observational period, 1060 newly diagnosed PD cases were identified. 4240 controls were randomly selected from those without PD using a matching strategy for age, sex, the year of PD diagnosis and the year of entering the NHI program at a ratio of 1:4. Ten elements of air pollution were examined, and multiple logistic regression models were used to measure their risks in subsequent PD development.

**Results:**

The incidence of PD in adults aged ≥ 40 years was 1.9%, and the median duration for disease onset was 8.45 years. None of the chemical compounds (SO_2_, O_3_, CO, NO_x_, NO, NO_2_, THC, CH_4_, or NMHC) significantly affected the incidence of PD except for particulate matter. PM_10_ exposure showed significant effects on the likelihood of PD development (T3 level: > 65μg/m^3^ versus T1 level: ≤ 54μg/m^3^; OR = 1.35, 95% CI = 1.12–1.62, 0.001 ≤ P < 0.01). In addition, comorbid conditions such as dementia (ORs = 3.53–3.93, Ps < 0.001), stroke (ORs = 2.99–3.01, Ps < 0.001), depression (ORs = 2.51–2.64, Ps < 0.001), head injury (ORs = 1.24–1.29, 0.001 ≤ Ps < 0.01 or 0.01 ≤ Ps < 0.05), sleep disorder (OR = 1.23–1.26, 0.001 ≤ Ps < 0.01), and hypertension (ORs = 1.18–1.19, 0.01 ≤ Ps < 0.05) also significantly increased the risk for PD development.

**Conclusions:**

Although PM_10_ plays a significant role in PD development, the associated chemical/metal compounds that are capable of inducing adverse biological mechanisms still warrant further exploration. Because of a link between comorbid conditions and PM exposure, research on the causal relationship between long-term exposure to PM and the development of PD should be considered with caution because other possible modifiers or mediators, comorbid diseases in particular, may be involved.

## Introduction

The detrimental effects of air pollution on human health have elicited great concern in recent decades. With the increasing number of empirical studies providing evidence on the contribution of chronic exposure to air pollution to the incidence of type 2 diabetes mellitus, cancers, and other diseases related to respiratory and cardiovascular functions [1–8], especially since these diseases may share common etiological mechanisms relative to neurodegeneration, the potential role of air pollution in the incidence of neurodegenerative disorders and its adverse impacts have become important issues in medicine and public health fields [9–17].

Parkinson’s disease (PD) is the second most prevalent neurodegenerative disorder after dementia in the elderly [18] and manifests with motor symptoms of bradykinesia, resting tremor, muscle rigidity and postural instability. Neuroinflammation has been revealed as a causal factor leading to the disease, and toxins in air pollution have been indicated to be capable of evoking a systemic inflammatory response and oxidative stress that can accelerate its pathology [19]. In addition, the preclinical period prior to the onset of PD in people at risk can last 5 to 10 years or even longer, and non-motor symptoms such as olfactory impairment, rapid eye movement sleep behavior disorder, anxiety, depression, and cardiovascular abnormalities may be present [20,21]. According to the WHO’s worldwide report, age-adjusted incidence rates range from 9.7 to 13.8 per 100,000 people per year. The prevalence is much higher, and the age-adjusted rates range from 72 to 258.8 per 100,000 persons [22]. As the population of the world ages and because PD has a long preclinical period, ascertaining the risk of air pollutants in subsequent PD development is important for the implementation of early interventions for people at risk and greater environmental protective strategies.

Abnormal aggregation of the α-synuclein protein, a major component of Lewy bodies, is believed to be a biomarker in the pathogenesis of PD. Animal models [23–24] and human studies [25–26] have demonstrated that air pollutants, including NO_2_, NO_x_, O_3_, and particulate matter (PM) in particular, are capable of inducing chronic inflammatory processes in the respiratory tract, passing the gastric epithelial lining, and breaking down the nasal/olfactory epithelial barrier and the blood-brain barrier to induce neurodegeneration in the brain as the associated inflammatory markers and the induced α-synuclein and/or amyloid β-42 were found. In addition, the chemical elements of lipopolysaccharides (LPS), manganese and carbon in PM are considered neurotoxic pathogens [26–29] in PD development. However, although evidence supports the deleterious effects of long-term exposure to air pollutants on PD pathology, a limited number of prospective studies in humans have shown inconsistent findings. Therefore, whether long-term exposure to air pollution could result in the development of PD remains ambiguous.

In addition to air-pollution exposure, aging, male gender, exposure to pesticides, brain injury, diabetes mellitus, vitamin D deficiency and sleep disturbances are risk factors for the incidence of PD and have been found to be positively associated with its development [30–36]. Contradictorily, other factors such as higher blood urate levels, the use of nonsteroidal anti-inflammatory drugs (NSAID), the use of calcium channel blockers, caffeine intake, alcohol consumption, and tobacco use have been found to be negatively associated with PD development [37–41]. The effects of using calcium channel blockers and NSAID drugs may be partially attributed to their links with comorbid conditions, such as cardiovascular diseases, since comorbid conditions may share biological pathways with air pollution toxins in PD. Taken together, in the present study, we conducted a population-based epidemiological study with a longitudinal design to examine the adverse effects of long-term exposure to air pollutants on the subsequent onset of PD with controlling for the potential risk factors.

## Materials and methods

### Data source

We utilized a database of 1,000,000 beneficiaries who were randomly sampled from the registration data of all beneficiaries enrolled in the Taiwan National Health Insurance Program (TNHIP) in the year 2000. Individual medical records were followed-up until the end of 2013, and the available records could be traced back to the beginning of TNHIP in the year 1995 in the National Health Insurance Research Database (NHIRD). This is a nationally representative sample managed and provided by the National Health Research Institutes (NHRI), which is authorized by the Ministry of Health and Welfare of Taiwan. The enrolled beneficiaries in the TNHIP occupy 99.9% of more than 23 million people in Taiwan, and the diseases of patients enrolled in the TNHIP are coded by clinical physicians who make diagnoses according to the criteria of the International Classification of Diseases, Ninth Revision, Clinical Modification (ICD-9-CM), which was introduced at the TNHIP’s commencement. Since the reimbursement processing is rigorous with professional peer reviews by a governmental single buyer-the National Health Insurance Administration (NHIA) and because misdiagnoses or miscoded diagnoses could lead to punitive action, the diagnostic codes that correspond to diseases are considered reliable. The NHIRD has the advantage of large-scale, longitudinal and reliable data, leading to extensive use. In addition, the NHIRD is confidential because all identification numbers of individuals have been anonymized. Therefore, informed consent from the sampled beneficiaries is not required for researchers to utilize the data.

We also utilized public data released from the Environmental Protection Administration (EPA) of Taiwan to obtain the concentrations of ambient pollutants from 76 monitoring stations across Taiwan during 1993–2016. This study was approved by the Institute Review Board of China Medical University Hospital (CMUH 104-REC2-115(CR-1)).

### Study design and measurement

We conducted a nested case-control study to examine the effects of long-term exposure to ambient air pollutants on subsequent onset of Parkinson’s disease in the studied population. The studied population included people living in the areas with monitoring stations established during 1995–1999 (N = 243,955) who were selected from the NHIRD. We excluded people younger than 40 years (N = 174,214) and patients diagnosed with Parkinson’s disease (ICD-9-CM: 332, N = 824) before the end of 1999. In addition, to establish a temporal relationship that the effects of exposure to air pollutants occurred earlier than the manifestation of comorbid diseases such as dementia, diabetes and stroke in particular, we excluded the patients diagnosed with these diseases (dementia: n = 243, diabetes: n = 9,259, stroke: n = 4,891) before January 1, 2000, which is the beginning of the observation period in this studied population.

**Fig 1:**
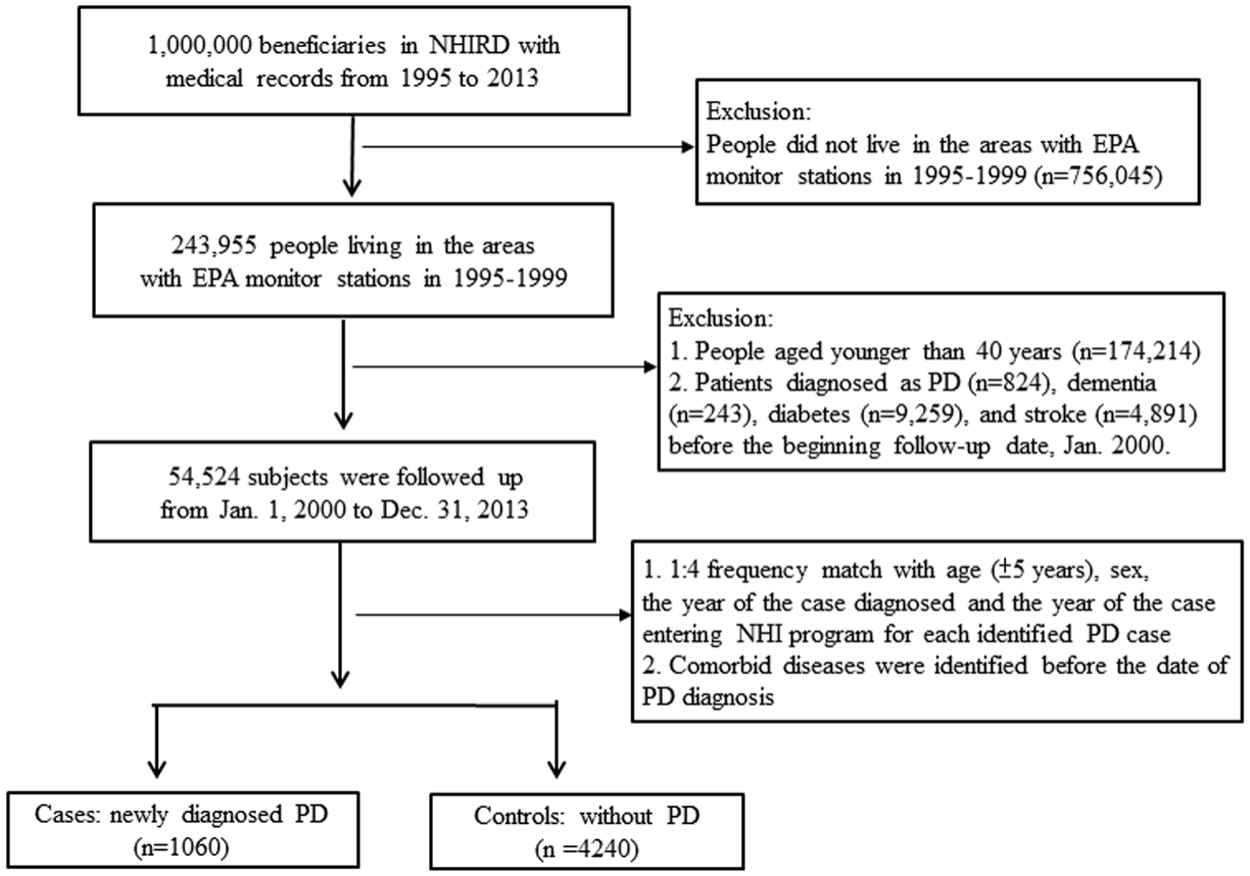
Study flow.

A total of 54,524 studied subjects were observed from January 1, 2000 to December 31, 2013. In clinical practice, the PD diagnosis was based on the findings from neurological examinations and imaging by well-trained physicians specialized in neurology. To increase the validity of clinical diagnoses, PD cases were identified from patients diagnosed with Parkinson's disease (ICD-9-CM: 322) in at least three outpatient visits within one year or in a one-time hospital admission. This definition was also used in studies that utilized the NHIRD [42, 43]. The control subjects were randomly selected from beneficiaries without PD using a matching strategy for age (±5 years), sex, the year of PD diagnosis and the year of NHI program entrance for each identified case. The ratio for selection was 1 to 4 (case: control). Over the course of 14 years of follow-up, we identified 1060 cases of PD and obtained 4240 controls (Fig 1).

Information is available in the NHIRD regarding other risk factors for PD. That is, medical histories on head injury (310.2, 800–801, 803–804, 850–854, and 959.01), sleep disorder (307, 327, and 780.5), vitamin D deficiency (268), diabetes (250), dementia (290, 294.1, and 331.0), stroke (430–438), hypertension (401–405), depression (296.2, 296.3, 300.4 and 311), tobacco use disorder (305.1), alcohol-related diseases (291, 303, 305, 571.0–571.3, 790.3 and V11.3), and renal disease (580–589) were identified before the diagnostic date of Parkinson’s disease. The above comorbidities were defined according to a diagnostic history in at least two outpatient visits within one year or a one-time hospital admission.

The monthly average ambient pollutants’ concentrations for each of the studied subjects (N = 5300) were calculated from the beginning of 1995 to December 31, 1999 across the EPA monitoring stations located in the areas that the subjects resided in during this period. Since the controls were randomly selected by matching with the year of entering the NIH program and the year of PD diagnosis, the time of exposure to ambient pollutants between the case and control groups was generally equal. This allowed us to examine the risk of the air pollutants’ concentrations for PD development by controlling for variations in exposure time across the groups. In the present study, we examined ten common air pollutants: sulfur dioxide (SO_2_), ozone (O_3_), carbon monoxide (CO), nitrogen oxides (NO_x_), nitrogen monoxide (NO), nitrogen dioxide (NO_2_), total hydrocarbons (THC), methane (CH_4_), non-methane hydrocarbons (NMHC), and PM_10_.

### Statistical analysis

To determine whether exposure to each selective ambient pollutant is a precursor to PD, we conducted simple and multivariate logistic regression models to estimate the risk of exposure relative to the subsequent occurrence of Parkinson’s disease. Adjusted odds ratios (ORs) were obtained by controlling for urbanization levels and comorbid diseases. In addition, since the distributions of the concentrations of air pollutants are skewed, tertile statistics were also used to depict their attributes and effects on the occurrence of PD. Before conducting the above analyses, a comparison of the characteristics between the cases and controls was completed using the χ2 test for categorical variables and a two-sample student T test for continuous variables. All statistical methods were performed using SAS version 9.3.

## Results

In the present study, a total of 54,524 subjects living in areas with 74 EPA monitoring stations across Taiwan in 1995–1999, with ages equal to or older than 40 years and without diagnosed PD, dementia, diabetes, or stroke before the end of 1999 were observed from Jan 1, 2000 to the end of 2013. During this period, 1060 subjects were diagnosed with Parkinson’s disease. In the studied subjects at the beginning of the observational time, the proportion of PD onset was 1.9%, and the incidence density over the follow-up period of 14 years was 1.56 per 1000 person-years.

The time for the onset of PD ranges from 1.07 to 13.94 years, and the median is 8.45 years. No differences were observed in the distributions of age, gender or urbanization level between the case and control groups (Table 1). The mean age at PD diagnosis in both groups was 72 years. Among the identified PD patients, the mean age at diagnosis for each age group—40–59, 60–69, 70–79 and 80+ years—were 54.2, 65.6, 75.0, and 83.9, respectively. Females were slightly more affected than males (51.8% > 48.2%). In PD patients, hypertension was the most prevalent comorbid disease (72.9%), followed by sleep disorder (51.0%), stroke (39.5%), head injury (21.4%) and depression (20.6%). For each comorbid disease, compared to those without the disease, the unadjusted risk for the subsequent development of PD was significantly higher than 1.0 except for tobacco use disorder. Dementia had the highest OR value of 5.43 (95% CI: 4.30–6.86), followed by stroke and depression (ORs: 4.00 and 3.50).

**Table 1:** Distributions of the demographic characteristics and the comorbid diseases between patients with and without Parkinson’s disease (PD) and their crude odds ratios.

Variable	With PD (N = 1060)	Without PD (N = 4020)	OR (95% CI)
	N (%)	N (%)	
Age^†^ diagnosed as PD (year)			
40–59	141 (13.3)	564 (13.3)	1
60–69	248 (23.4)	992 (23.4)	1.00(0.79–1.26)
70–79	420 (39.6)	1680 (39.6)	1.00(0.81–1.24)
80+	251 (23.7)	1004 (23.7)	1.00(0.79–1.26)
Gender			
Women	549 (51.8)	2196 (51.8)	1
Men	511 (48.2)	2044 (48.2)	1.00 (0.87–1.14)
Urbanization level			
5 (lowest)	143 (13.5)	600 (14.2)	1
4	207 (19.5)	714 (16.8)	1.22 (0.96–1.55)
3	170 (16.0)	723 (17.1)	0.99 (0.77–1.26)
2	281 (26.5)	1109 (26.2)	1.06 (0.85–1.33)
1 (highest)	259 (24.4)	1094 (25.8)	0.99 (0.79–1.25)
Comorbid disease (ref^#^: no)			
Hypertension	773 (72.9)	2631 (62.0)	1.65 (1.42–1.91)***
Diabetes	211 (19.9)	674 (15.9)	1.32 (1.10–1.56)**
Stroke	419 (39.5)	595 (14.0)	4.00 (3.45–4.65)***
Depression	218 (20.6)	292 (6.89)	3.50 (2.89–4.24)***
Renal disease	182 (17.2)	571 (13.5)	1.33 (1.11–1.60)**
Dementia	171 (16.1)	145 (3.42)	5.43 (4.30–6.86)***
Head injury	227 (21.4)	566 (13.3)	1.77 (1.49–2.10)***
Alcohol-related disease	29 (2.74)	51 (1.2)	2.31 (1.46–3.66)***
Tobacco use disease	23 (2.17)	60 (1.4)	1.55 (0.95–2.51)
Sleep disorder	541 (51.0)	1596 (37.6)	1.73 (1.51–1.98)***

^†^: Mean (SD): 72.2 (9.96) in PD and 72.1 (9.96) in non-PD; the mean duration of the subsequent onset of PD since the beginning of the follow-up is 8.12 (3.54) years;

^#^ indicates that the reference group for each comorbid disease's OR is the group without the disease;

*: P < 0.05;

**: P < 0.01;

***: P < 0.001.

No significant differences existed in the distributions of the monthly average cumulative exposure to each of the ambient pollutants between both groups, except for PM_10_, where the subjects with PD were exposed to higher PM_10_ concentrations than their counterparts (mean (SD): 62.3 (13.8) > 60.6 (13.6) μg/m^3^, P = .0004, table not shown). The estimated odds ratios for the later development of PD for each of the pollutants, assessed by either continuous or tertile quantities without controlling for age, urbanization levels, and comorbid diseases, showed similar results with those obtained from the multivariate analyses (Table 2). Still, only PM_10_ exposure showed significant effects on the likelihood of PD development (T3 level versus T1 level: OR = 1.35, 95% CI = 1.12–1.62, 0.001 ≤ P < 0.01; per unit increase in PM_10_ (μg/m^3^) would increase the likelihood of having PD 1.01 times, P < 0.001), indicating that compared to people exposed to PM_10_ concentration levels less than or equal to 54 μg/m^3^ (1^st^ tertile is at 54; T1 level: ≤54), people exposed levels up to above 65 μg/m^3^ (2^nd^ tertile is at 65; T3 level: > 65; T2 level: > 54, ≤ 65) would have a 1.35 times higher risk for the subsequent development of PD.

**Table 2:** The adjusted odds ratios (95% CIs) of the exposure to each air pollutant and the associated risk factors for Parkinson’s disease.

Variable	SO_2 (ppb)_	O_3 (ppb)_	CO _(ppm)_	NO_x (ppb)_	NO _(ppb)_
Air pollution level (ref: T1)					
T2	1.06 (0.89–1.26)	1.22 (0.99–1.48)	0.99 (0.81–1.20)	0.91 (0.75–1.10)	0.91 (0.75–1.10)
T3	1.11 (0.92–1.35)	1.16 (0.94–1.43)	1.00 (0.81–1.23)	0.95 (0.77–1.18)	0.95 (0.77–1.18)
Per 1 unit increase	1.20 (0.99–1.05)	1.01 (0.97–1.04)	0.99 (0.60–1.64)	1.00 (0.99–1.01)	0.99 (0.98–1.01)
Urbanization level (ref: level 5)					
1 (highest)	1.01 (0.79–1.30)	1.01 (0.78–1.30)	1.04 (0.78–1.37)	1.07 (0.80–1.43)	1.07 (0.80–1.43)
2	1.05 (0.82–1.35)	1.08 (0.85–1.38)	1.09 (0.84–1.40)	1.12 (0.87–1.45)	1.12 (0.87–1.45)
3	1.02 (0.78–1.35)	1.08 (0.83–1.41)	1.07 (0.80–1.42)	1.12 (0.84–1.48)	1.12 (0.84–1.48)
4	1.29 (0.99–1.67)	1.32 (1.02–1.70)*	1.31 (1.01–1.70)*	1.33 (1.03–1.73)*	1.33 (1.03–1.73)*
Comorbid disease (ref: no)					
Hypertension	1.18 (1.00–1.40)*	1.19 (1.00–1.40)*	1.18 (1.00–1.39)*	1.18 (1.00–1.39)*	1.18 (1.00–1.39)*
Diabetes	1.04 (0.86–1.26)	1.04 (0.86–1.25)	1.04 (0.86–1.26)	1.04 (0.86–1.26)	1.04 (0.86–1.26)
Dementia	3.55 (2.76–4.57)***	3.53 (2.74–4.54)***	3.54 (2.75–4.56)***	3.54 (2.75–4.55)***	3.54 (2.75–4.55)***
Stroke	3.00 (2.54–3.53)***	3.01 (2.55–3.54)***	3.00 (2.54–3.53)***	3.00 (2.55–3.54)***	3.00 (2.55–3.54)***
Depression	2.53 (2.05–3.13)***	2.54 (2.05–3.13)***	2.53 (2.05–3.13)***	2.53 (2.05–3.12)***	2.53 (2.05–3.12)***
Renal disease	1.00 (0.82–1.22)	1.00 (0.81–1.22)	1.00 (0.82–1.22)	1.00 (0.82–1.22)	1.00 (0.82–1.22)
Sleep disorder	1.24 (1.06–1.44)**	1.25 (1.07–1.45)**	1.24 (1.06–1.44)**	1.24 (1.06–1.44)**	1.24 (1.06–1.44)**
Alcohol-related disease	1.51 (0.90–2.52)	1.52 (0.91–2.54)	1.51 (0.91–2.52)	1.51 (0.90–2.52)	1.51 (0.90–2.52)
Head injury	1.29 (1.07–1.56)**	1.29 (1.07–1.56)**	1.29 (1.07–1.56)**	1.29 (1.07–1.56)**	1.29 (1.07–1.56)**
Variable	NO_2 (ppb)_	THC _(ppm)_	CH_4 (ppm)_	NMHC _(ppm)_	PM_10 (μg/m_^3^_)_
Air pollution level (ref: T1)					
T2	1.03 (0.84–1.25)	1.06 (0.88–1.28)	0.92 (0.75–1.12)	0.87 (0.71–1.07)	1.17 (0.98–1.41)
T3	1.04 (0.85–1.28)	1.03 (0.84–1.27)	1.13 (0.94–1.35)	0.88 (0.68–1.12)	1.35 (1.12–1.62)**
Per 1 unit increase	1.01 (0.99–1.03)	1.02 (0.56–1.86)	1.65 (0.66–4.14)	0.64 (0.26–1.56)	1.01 (1.00–1.02)***
Urbanization level (ref: level 5)					
1 (highest)	1.01 (0.76–1.33)	1.00 (0.76–1.31)	1.07 (0.82–1.40)	1.11 (0.83–1.48)	1.14 (0.88–1.47)
2	1.07 (0.83–1.38)	1.08 (0.83–1.41)	1.14 (0.88–1.48)	1.16 (0.89–1.53)	1.15 (0.90–1.47)
3	1.05 (0.80–1.38)	1.04 (0.77–1.40)	1.08 (0.80–1.46)	1.11 (0.82–1.52)	1.11 (0.85–1.45)
4	1.31 (1.01–1.69)*	1.39 (1.04–1.84)*	1.42 (1.06–1.88)*	1.40 (1.05–1.86)*	1.35 (1.04–1.75)*
Comorbid disease (ref: no)					
Hypertension	1.18 (1.00–1.39)*	1.18 (0.99–1.41)	1.18 (0.99–1.41)	1.18 (0.99–1.41)	1.18 (1.00–1.40)*
Diabetes	1.04 (0.86–1.26)	1.05 (0.86–1.28)	1.05 (0.86–1.28)	1.05 (0.86–1.29)	1.04 (0.87–1.26)
Dementia	3.54 (2.75–4.56)***	3.92 (2.98–5.16)***	3.93 (2.99–5.17)***	3.93 (2.99–5.17)***	3.55 (2.76–4.56)***
Stroke	3.00 (2.55–3.54)***	3.00 (2.51–3.57)***	3.00 (2.52–3.58)***	2.99 (2.51–3.57)***	3.01 (2.56–3.55)***
Depression	2.53 (2.05–3.13)***	2.63 (2.10–3.30)***	2.64 (2.10–3.30)***	2.63 (2.10–3.30)***	2.51 (2.03–3.10)***
Renal disease	1.00 (0.82–1.22)	1.00 (0.80–1.24)	1.00 (0.80–1.23)	1.00 (0.81–1.24)	0.99 (0.81–1.21)
Sleep disorder	1.23 (1.06–1.44)**	1.25 (1.06–1.47)**	1.26 (1.07–1.48)**	1.25 (1.06–1.47)**	1.24 (1.06–1.44)**
Alcohol-related disease	1.51 (0.91–2.53)	1.69 (0.97–2.93)	1.67 (0.97–2.89)	1.69 (0.97–2.92)	1.49 (0.89–2.48)
Head injury	1.29 (1.07–1.56)**	1.25 (1.02–1.53)*	1.24 (1.01–1.52)*	1.25 (1.02–1.54)*	1.28 (1.06–1.55)**

Each model included only one air pollutant controlled for age, sex, urbanization level, and comorbid disease.

*: P < 0.05

**: P < 0.01

***: P < 0.001

With regard to the examined effects of other risk factors on the occurrence of PD across all analytic models in Table 2, we found that compared to people living in the area with the lowest urbanization level, people living in the areas with slightly better urbanization levels would encounter a 1.31 to 1.42 times higher risk for developing PD (ORs = 1.31–1.42, 0.01 ≤ Ps < 0.05). The estimated effects of comorbid diseases with controlling for each air pollutant indicated that having a diagnosis of dementia (ORs = 3.53–3.93, Ps < 0.001), stroke (ORs = 2.99–3.01, Ps < 0.001), depression (ORs = 2.51–2.64, Ps < 0.001), head injury (ORs = 1.24–1.29, 0.001 ≤ Ps < 0.01 or 0.01 ≤ Ps < 0.05), sleep disorder (OR = 1.23–1.26, 0.001 ≤ Ps < 0.01), or hypertension (ORs = 1.18–1.19, 0.01 ≤ Ps < 0.05) would increase the risk for developing PD, but the hypertension effect was not significant for the models with THC, CH_4_ or NMHC. The significant associations of diabetes, renal disease, and alcohol-related disease in the bivariate analyses became disappeared.

**Fig 2:**
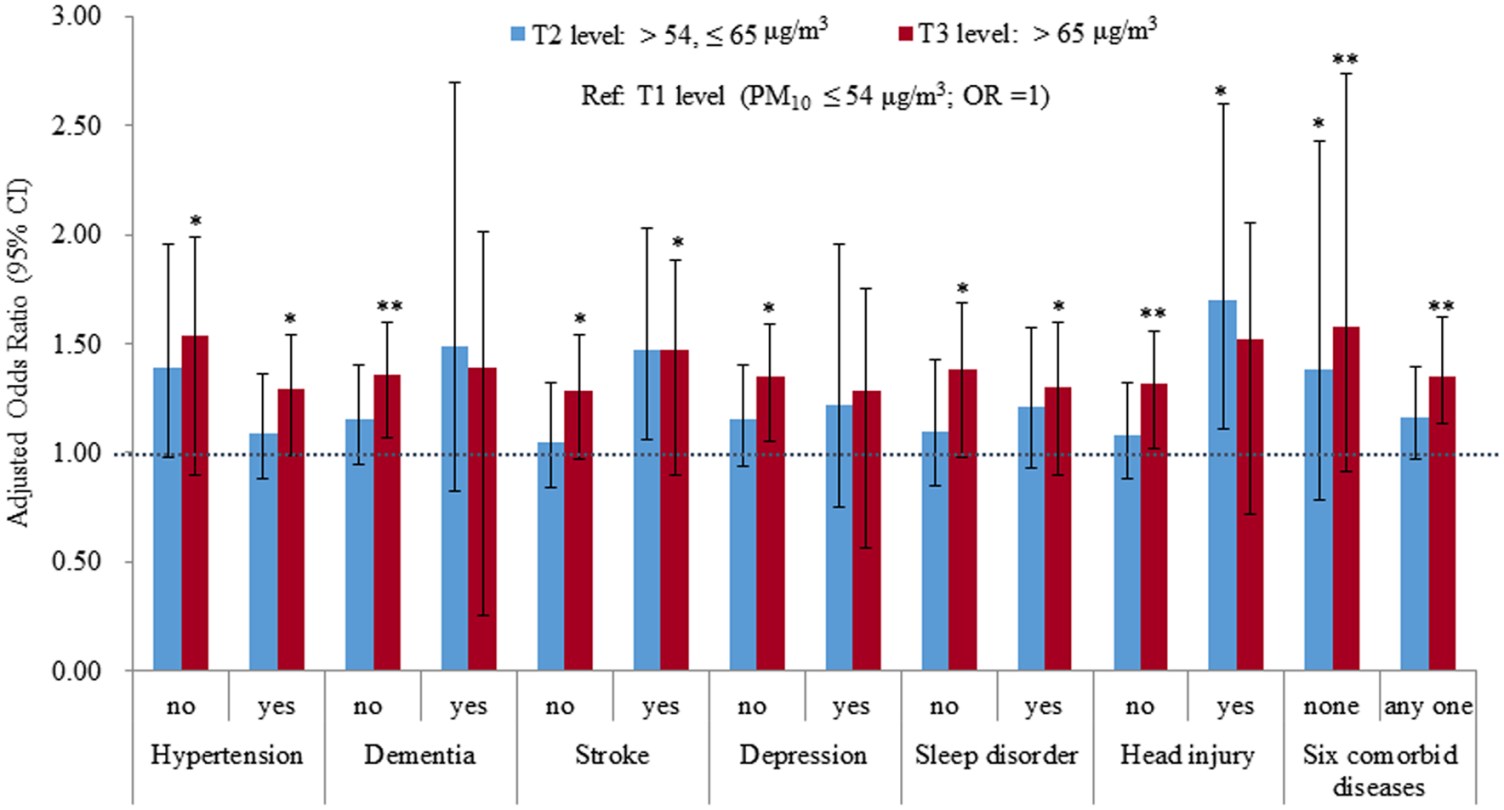
The adjusted odds ratios of the exposure to PM_10_ and a later PD onset by comorbid diseases. Ref: T1 level (PM_10_ ≤ 54 μg/m^3^; OR = 1). *: P < 0.05; **: P < 0.01; ***: P < 0.001.

We conducted stratified analyses by subjects with and without any one of the six comorbid diseases that were identified as significant in the previous analysis, and also conducted stratified analyses by subjects with any one of the six diseases and without any of them to examine the effects of PM_10_ exposure on the incidence of PD. The results across the multiple logistic regression models for each of the six diseases indicated that the effect of PM_10_ exposure on PD onset was not significant for subjects with dementia (N = 316) or for subjects with depression (N = 501). The relationship of PM_10_ exposure with a later PD onset was significant in subjects with the other four diseases and in subjects without any of the six diseases (Fig 2).

In addition to PM_10_, the adverse effects of the other comorbid diseases were also evident in each of the subgroup analyses (table not shown). In the subjects with dementia and with depression, the insignificant effects of PM_10_ may be due to the stronger contributions to the risk of PD development by comorbid stroke (OR = 1.61, P < 0.05) and comorbid dementia (OR = 2.49, P < 0.001), respectively. However, in contrast to the significant effect of PM_10_ exposure in the subjects with any comorbid disease (N = 4340), an insignificant effect was found in subjects without any comorbid disease (N = 960) (Table 3). Finally, a multiple logistic regression model was conducted for all subjects. The results in Table 3 showed a significantly positive effect of long-term exposure to PM10 on subsequent PD development regardless of the effect of comorbid conditions (T3 level versus T1 level: OR = 1.37, P < 0.001), and the pre-existence of any comorbid diseases would increase the likelihood for the incidence of PD (OR = 2.83, P < 0.001).

**Table 3:** The adjusted odds ratios (95% CIs) of PM10 exposure for a later PD onset in subjects with and without any comorbid diseases, and in all subjects.

Variable	without comorbid diseases	with comorbid diseases	all subjects
	(N = 960)	(N = 4340)	(N = 5300)
Air pollution level (ref: T1)			
T2	1.38 (0.78–2.43)	1.16 (0.97–1.39)	1.18 (0.99–1.40)
T3	1.58 (0.91–2.74)	1.35 (1.13–1.62)**	1.37 (1.15–1.62)***
Per 1 unit increase	1.01 (0.99–1.03)	1.01 (1.00–1.01)**	1.01 (1.00–1.01)**
Urbanization level (ref: level 5)			
1 (highest)	0.90 (0.41–1.99)	1.15 (0.90–1.47)	1.05 (0.83–1.33)
2	1.25 (0.59–2.63)	1.13 (0.89–1.44)	1.09 (0.86–1.37)
3	0.70 (0.30–1.64)	1.11 (0.85–1.44)	1.00 (0.78–1.29)
4	1.51 (0.68–3.33)	1.23 (0.95–1.59)	1.23 (0.96–1.57)
Comorbid diseases (ref: none)			
With one or more			2.83 (2.24–3.57)***

Each model included age and sex, and their effects were insignificant.

Comorbid diseases included ten diseases as shown in Table 1.

*: P < 0.05

**: P < 0.01

***: P < 0.001

## Discussion

Our findings indicated that particulate matter, PM_10_, significantly contributed to the incidence of PD, but the remaining nine pollutants (SO_2_, O_3_, CO, NO_x_, NO, NO_2_, THC, CH_4_, and NMHC) did not. Air pollution, as described in detail previously [19], contains a complex mixture of gases (e.g., ozone, carbon monoxide, sulfur oxides, and nitrogen oxides), particulate matter, organic compounds (e.g., polycyclic aromatic hydrocarbons and endotoxins) and metals (e.g., vanadium, nickel, and manganese). With this complexity, it becomes challenging for one to understand the specific chemical compounds in air pollution and their potential causal links to specific medical conditions. Nevertheless, to our knowledge, among the air pollutants, PM and NO_2_ were salient and were found to be associated with the risk of PD in the existing empirical studies [13–17, 44]. Although both culprits were found in these studies, not all studies identified both at the same time. This inconsistency may be due in part to the variations in the study designs and the measurements of the exposure to air pollutants across studies. However, we found an insignificant effect of NO_2_, which is consistent with the results in a recent study with a similar design to ours [45].

PM is a component of air pollution that is made up of extremely small particles and liquid droplets containing acids, organic chemicals, metals, and soil or dust particles [46] and is categorized into coarse (PM_10_), fine (PM_2.5_), and ultrafine (PM_0.1_) based on mean particle size fractions less than 10, 2.5, and 0.1 μm in aerodynamic diameter, respectively. All PMs can be carriers for additional air pollutant compounds such as polyaromatichydrocarbons and elemental metals. The sources of PM_10_ include road and agricultural dust, tire wear emissions, wood combustion, construction and demolition work, and mining operations [47]. The major sources of PM_2.5_ include oil refineries, metal processing facilities, tailpipe and brake emissions from mobile sources, residential fuel combustion, power plants, and wildfires. The primary sources of ultrafine PM include tailpipe emissions from mobile sources [48].

The study of PM has encountered challenges for understanding its biological effects due to its physical and chemical complexity. However, recent studies have suggested that among a variety of air pollutants, PM is a particularly important contributor to CNS diseases. Stroke, dementia, Alzheimer’s disease and Parkinson’s disease have been studied, but the studies are few, and the evidence appears contradictory [8–9, 11–17]. The pathological mechanism of these diseases relative to air pollution is well-proposed, that is, the toxins in air pollution induce inflammation, reactive oxygen species (ROS), and neuropathology that incite the central nervous system. PM toxicity can lead not only to brain cytokine production through respiratory tract inflammation and transportation in the systemic circulation via macrophage-like cells but also to nasal respiratory and olfactory damage, one origin of PD pathology. This pathway has been supported by some empirical studies with animal and human subjects [49–53].

Our study has several strengths. First, it is a large-scale human study with a national representative sample, and the size of the studied cohort, ages ≥ 40 years (N = 54,524), is large enough to detect cases of later PD onset and consider the heterogeneity among the risk factors for examining their odds ratios. Second, the 14-year follow-up period covers the 5- to 10-year preclinical period of PD. Indeed, our study indicates a median duration of 8.45 years for PD onset. Third, we excluded any subjects diagnosed with PD before the observational period beginning on Jan 1, 2000 and assessed the 5-year exposure to air pollution prior to the start date to establish a better temporal relationship. This design is unlike those in most studies in which assessments of exposure to air pollution often covered the observational period, which was from the beginning of the study to the endpoint of PD onset. This design could make the causal link between air-pollution exposure and the incidence of PD more difficult because the onset of PD could be attributed more to the complications of comorbid diseases, as the inflammatory processes induced by air pollution may lead to the occurrence of comorbid diseases earlier than PD. To avoid interference from the concurrent existence of comorbid diseases and air-pollution exposure, we did not consider the exposure to air pollution in the follow-up period and excluded some comorbid diseases thought to have strong associations with PD, including dementia, stroke and diabetes that were diagnosed before the beginning date. Fourth, the present study is a nested case-control study, and the use of the matching design to obtain the controls-matching by the year that the PD case entered the NHI program, the year of PD diagnosis, age and sex-can control for the confounding effects of age, sex and the time length of the exposure to air pollution between those with PD and those without. The same duration of air-pollution exposure between both groups allows us to examine only the variations in the concentrations of air pollution exposure between them.

There are some limitations in the present study. First, there is a lack of data on related biomarkers or risk factors such as blood urate levels, the use of NSAIDs and calcium channel blockers, caffeine intake, exposure to metals and pesticides, and family medical histories or genetic information. Second, the cases were identified by ICD-9-CM codes, and a diagnostic bias may have occurred. To ensure the accuracy of identifying cases, we restricted the diagnosis of PD to at least three outpatient visits within one year or a one-time hospitalization. Nevertheless, an overestimation may occur for the associations of comorbid diseases due to a possible attendance bias because of the greater likelihood of having subsequent diagnoses in those with an initial diagnosis. Third, we did not examine the effects of fine particulate matter because the EPA in Taiwan did not monitor these concentrations until 1998, and the two-year exposure to PM_2.5_ during 1998–1999 would limit us in the examination of its detrimental effects. However, our study results support the temporal link between long-term exposure to PM_10_ and the incidence of PD to some extent, which may infer the possible effects of PM_2.5_ as both pollutants may correlate to each other.

## Conclusions

In this nested case-control study, we found that among air pollutants, PM_10_ plays a significant role in the development of Parkinson’s disease. Although the PM-associated chemical/metal compounds capable of inducing the adverse biological mechanisms that lead to the occurrence of PD require further exploration, the results in our analyses suggested a comorbidity effect. Therefore, research that examines the causal relationship between long-term exposure to PM and PD should be cautious and must consider possible modifiers or mediators, especially comorbid diseases. The possible modifying or intermediating effects of comorbid diseases on this relationship could help in the further understanding of the underlying pathways in which the toxicity of air pollution exposure affects the development of neurodegenerative disorders.
